# Phase 2 study of preoperative chemotherapy with nab‐paclitaxel and gemcitabine followed by chemoradiation for borderline resectable or node‐positive pancreatic ductal adenocarcinoma

**DOI:** 10.1002/cam4.5971

**Published:** 2023-05-03

**Authors:** Emerson Y. Chen, Adel Kardosh, Nima Nabavizadeh, Bryan Foster, Skye C. Mayo, Kevin G. Billingsley, Erin W. Gilbert, Christian Lanciault, Aaron Grossberg, Kenneth G. Bensch, Erin Maynard, Eric C. Anderson, Brett C. Sheppard, Charles R. Thomas, Charles D. Lopez, Gina M. Vaccaro, OHSU Gastrointestinal Cancer Disease Group

**Affiliations:** ^1^ Division of Hematology and Medical Oncology Oregon Health & Science University, Knight Cancer Institute Portland Oregon USA; ^2^ Department of Radiation Medicine Oregon Health & Science University Portland Oregon USA; ^3^ Department of Diagnostic Radiology Oregon Health & Science University Portland Oregon USA; ^4^ Division of Surgical Oncology Oregon Health & Science University, Knight Cancer Institute Portland Oregon USA; ^5^ Yale Cancer Center New Haven Connecticut USA; ^6^ Division of Gastrointestinal and General Surgery Oregon Health & Science University Portland Oregon USA; ^7^ Providence Cancer Institute Portland Oregon USA; ^8^ Portland VA Medical Center Portland Oregon USA; ^9^ Radiation Oncology Geisel School of Medicine at Dartmouth and Dartmouth Cancer Center New Hampshire Lebanon USA

**Keywords:** chemoradiation, clinical trial, gemcitabine, nab‐paclitaxel, neoadjuvant, pancreatic cancer

## Abstract

**Background:**

Neoadjuvant treatment with nab‐paclitaxel and gemcitabine for potentially operable pancreatic adenocarcinoma has not been well studied in a prospective interventional trial and could down‐stage tumors to achieve negative surgical margins.

**Methods:**

A single‐arm, open‐label phase 2 trial (NCT02427841) enrolled patients with pancreatic adenocarcinoma deemed to be borderline resectable or clinically node‐positive from March 17, 2016 to October 5, 2019. Patients received preoperative gemcitabine 1000 mg/m^2^ and nab‐paclitaxel 125 mg/m^2^ on Days 1, 8, 15, every 28 days for two cycles followed by chemoradiation with 50.4 Gy intensity‐modulated radiation over 28 fractions with concurrent fluoropyrimidine chemotherapy. After definitive resection, patients received four additional cycles of gemcitabine and nab‐paclitaxel. The primary endpoint was R0 resection rate. Other endpoints included treatment completion rate, resection rate, radiographic response rate, survival, and adverse events.

**Results:**

Nineteen patients were enrolled, with the majority having head of pancreas primary tumors, both arterial and venous vasculature involvement, and clinically positive nodes on imaging. Among them, 11 (58%) underwent definitive resection and eight of 19 (42%) achieved R0 resection. Disease progression and functional decline were primary reasons for deferring surgical resection after neoadjuvant treatment. Pathologic near‐complete response was observed in two of 11 (18%) resection specimens. Among the 19 patients, the 12‐month progression‐free survival was 58%, and 12‐month overall survival was 79%. Common adverse events were alopecia, nausea, vomiting, fatigue, myalgia, peripheral neuropathy, rash, and neutropenia.

**Conclusion:**

Gemcitabine and nab‐paclitaxel followed by long‐course chemoradiation represents a feasible neoadjuvant treatment strategy for borderline resectable or node‐positive pancreatic cancer.

## 1 INTRODUCTION

U.S. mortality from pancreatic cancer is expected to be 50,550 annually in 2023, accounting for 8% of all cancer‐related deaths.^1^ Multiple clinical trials and preclinical studies have demonstrated that even when initial evaluation suggests localized disease, pancreatic adenocarcinoma has a high potential for systemic spread to the liver, lungs, and peritoneum.^2^ Multiagent systemic chemotherapy therefore is vital to eliminate micro‐metastases and improve overall outcome. One common regimen, 5‐fluorouracil, leucovorin, oxaliplatin, and irinotecan (FOLFIRINOX) has demonstrated cure rates of up to 40% when incorporated as adjuvant therapy for very fit patients after successful resection.^3^ Gemcitabine with capecitabine or nab‐paclitaxel likewise has emerged as clinically active adjuvant regimens, which decrease the risk of disease recurrence after successful surgical resection.^4^, ^5^ However, it remains unclear how long‐term survival from these regimens would compare to FOLFIRINOX.

Neoadjuvant therapy has been readily adopted by many institutions in recent years in the U.S., especially for borderline resectable patients who are at higher risk of positive surgical margins and future disease relapse.^6^ Neoadjuvant therapy, including both multiagent chemotherapy and concurrent chemoradiation, serves as a means to eradicate microscopic disease early and down‐stage tumors to better achieve negative surgical margins. In fact, two meta‐analyses gathering retrospective, prospective, and interventional trials globally have now accumulated clear evidence of at least comparable, if not superior, overall survival when starting with neoadjuvant chemotherapy or chemoradiation instead of immediate surgery for both resectable and borderline resectable patients with pancreatic ductal adenocarcinoma (PDAC).^7^, ^8^ Additional randomized head‐to‐head trials comparing neoadjuvant to adjuvant strategy are underway. However, the optimal preoperative chemotherapy and radiation regimens, whether in sequence or concurrent, have not yet been specifically established prospectively.

FOLFIRINOX, gemcitabine with capecitabine, and concurrent gemcitabine or fluoropyrimidine with radiation have all been evaluated as potential neoadjuvant treatment strategies in phase 2 or 3 trials for patients with borderline resectable PDAC, noting resection rates of 55%–67% and final margin‐negative (R0) resection of 23%–65%.^9^, ^10^, ^11^ How these regimens are used in real‐world practice currently depends on multidisciplinary conferences to confirm resectability and estimate the risk of metastatic disease to give the best treatment sequence.^12^ However, specific clinical outcomes of nab‐paclitaxel with gemcitabine in the neoadjuvant chemotherapy‐radiation setting have not been well studied, but we hypothesize the R0 resection would be as good as, if not better than, the historically observed proportions from other regimens. A recent randomized trial noted similar R0 resection rate as FOLFIRINOX.^13^, ^14^

Therefore, we conducted a phase 2 trial using nab‐paclitaxel and gemcitabine followed by chemoradiation with fluoropyrimidine to test the feasibility and clinical outcomes of this novel neoadjuvant regimen in patients with borderline resectable and clinically node‐positive PDAC at our institution. We anticipated the proposed neoadjuvant treatment would increase R0 resection to 56% from a prior historical rate of 37%, which was a lower‐limit estimate among published neoadjuvant trials.^9^, ^10^, ^11^

## 2 METHODS

### 2.1 Participants

All adults with newly diagnosed, biopsy‐proven PDAC were eligible if they were deemed to be borderline resectable or clinically node‐positive at our multidisciplinary case conference. The borderline resectable criteria were defined per American Hepato‐Pancreato‐Biliary Association, Society of Surgical Oncology, and Society for Surgery of the Alimentary Tract consensus criteria with arterial and/or venous involvement of celiac, mesenteric, and/or portal vasculature.^15^ Node‐positive PDAC was defined if abnormal regional lymph nodes were visible on contrast computed tomography (CT). Lymph node biopsy and PET evaluation were not required. Node‐positive PDAC was included in our high‐risk study population because an increased number of positive lymph nodes have high rates of disease recurrence and poor survival.^16^, ^17^ Retro‐peritoneal lymph nodes were deemed as metastatic disease at our institution. All participants had adequate functional status, acceptable organ and bone marrow function, unremarkable co‐morbidities, and minimal to null baseline neuropathy to be able to undergo multimodal neoadjuvant therapy. Details of the inclusion and exclusion are detailed in public website (https://clinicaltrials.gov/ct2/show/NCT02427841). Resectable node‐negative, unresectable locally advanced, and metastatic PDAC were all excluded. Active infections, concurrent cancers, interstitial lung disease, and prior treatment with chemotherapy, radiation, or surgery to the pancreas likewise were excluded.

### 2.2 Study design

Oregon Health & Science University (OHSU) Gastrointestinal Cancer Disease Group undertook a single‐arm, open‐label phase 2 trial (NCT02427841) in Portland, Oregon, enrolling patients from March 17, 2016 to October 5, 2019. Patients were scheduled to initially receive preoperative chemotherapy with gemcitabine 1000 mg/m^2^ and nab‐paclitaxel 125 mg/m^2^ on Days 1, 8, 15 every 28 days for two cycles, with dose‐schedule consistent with the FDA‐approved package insert.^18^

If patients experienced no disease progression radiographically following 2 months of chemotherapy, they then proceeded with preoperative chemoradiation with 50.4 Gy intensity‐modulated radiation therapy over 28 daily fractions of 1.8 Gy per fraction. Radiation therapy treatment volumes consisted of the primary tumor and clinically involved regional adenopathy >10 mm on the baseline (prechemotherapy) imaging (i.e., gross tumor volume [GTV]). No nodal regions were irradiated prophylactically. An additional 10 mm expansion was utilized on the GTV to encompass microscopically involved disease (clinical target volume). Finally, a 20 mm cranial‐caudal expansion and 10 mm axial expansion were utilized to account for respiratory motion and set‐up inaccuracies (planning target volume). Daily image guidance with cone‐beam CT was performed. Patients received concurrent IV 5‐fluorouracil 225 mg/m^2^/day for 5 or 7 days, or PO capecitabine 825 mg/m^2^ twice daily given Monday–Friday during each week of radiation.

If no evidence of metastatic disease was noted following neoadjuvant chemotherapy and chemoradiation, patients subsequently underwent definitive resection within 4–12 weeks followed by an additional four cycles of gemcitabine and nab‐paclitaxel postoperatively upon clinical recovery. Study schema is graphically presented in Figure S1. Dose modification scheme of gemcitabine and nab‐paclitaxel is provided in Table S1.

This study was done in accordance with the Declaration of Helsinki and was approved by local institutional review board at OHSU. All participants completed written informed consent prior to initiating any research procedures.

### 2.3 Assessments

The primary endpoint was margin‐negative (R0) resection, defined by clear surgical margins of at least 1 mm, among all enrolled patients.^19^ Proportions of patients completing neoadjuvant therapy and undergoing definitive surgery were also outcomes of interest. Pathologic response was graded per a validated tumor regression grading scale.^20^

All participants had contrast‐enhanced CT chest, abdomen, and pelvis completed at baseline, many of whom had dedicated multiphase pancreas protocol, but this was not required per protocol. Subsequent contrast‐enhanced CT scans were obtained after two cycles of gemcitabine and nab‐paclitaxel, after chemoradiation, and after surgery before subsequent four cycles of chemotherapy. Radiographic response was evaluated using revised Response Evaluation Criteria in Solid Tumors (RECIST) guideline (version 1.1),^21^ with the best radiographic response reported for either assessment prior to resection. Carbohydrate antigen (CA) 19‐9 levels were also collected at baseline before and during chemotherapy and chemoradiation. The Common Terminology Criteria for Adverse Events version 4.0 was used to record all toxicities from chemotherapy and radiation.

All participants had at least 1‐year follow‐up after surgery if they were still alive and remained in the clinical trial. After completion of all therapies, patients underwent surveillance with CA19‐9 and contrast CT scans approximately every 3 months until an event of interest or end of follow‐up. Many patients had longer follow‐up.

### 2.4 Statistical analysis

All 19 participants received at least two cycles neoadjuvant therapy and one efficacy assessment and were eligible for the efficacy analyses. We had hypothesized that, with our proposed neoadjuvant treatment, the R0 resection would increase to 56% from a prior historical proportion of 37%. Forty‐four evaluable patients were required to achieve 83% power using a 2‐sided binomial test at 10% significance level. The Simon's two‐stage minimax design was used based on the null and alternative hypothesis (37% vs. 56%). For the first‐stage, 19 subjects were required for interim analysis, and the trial would be stopped if 7 or fewer subjects underwent R0 resection. If 8 or more R0 resections were achieved, then the study would proceed until the intended sample size of 44. Time‐to‐event endpoints were estimated using the Kaplan–Meier method and report an associated 95% confidence interval (CI). Progression‐free survival (PFS) was measured from the first dose of study drug until progression or death, censoring at the time of last disease assessment. Among the resected, relapse‐free survival was estimated from the date of resection until progression or death, censoring at the time of last disease assessment. Survival was measured from the first dose of study drug until death, censoring at the date of last contact. Response evaluations were reported as proportions with 95% CIs calculated using the binomial exact method. Baseline characteristics were reported as frequency and percent (categorical variables) or median and range (minimum and maximum, continuous variables). All data were analyzed using SAS version 9.4 (SAS Institute Inc.).

## 3 RESULTS

### 3.1 Baseline characteristics

From March 2016 to October 2019, 24 patients were screened, but four were ineligible and one withdrew consent during screening. Nineteen patients were enrolled; they were all followed until death or data cutoff date, June 1, 2021. Despite meeting the interim standard to continue to second stage of the trial, the study ended early following a multidisciplinary review at OHSU and discussion with the sponsor in consideration of other ongoing national neoadjuvant trials, which would impact timely accrual. Among the 19 patients, the median age was 68 (range 42–76). The majority had Eastern Cooperative Oncology Group performance status 1, head of pancreas primary tumor, involvement of both arterial and venous vasculature, clinically positive nodes, and abnormally elevated CA19‐9 at baseline (Table 1). Four patients had normal CA19‐9 at baseline. Two patients did have resectable disease with no vascular involvement but had clinically positive nodes, thus eligible for the study, after multidisciplinary consensus.

**TABLE 1:** Baseline characteristics.

*N* = 19	Counts (%)
Age	68 (42–76)^a^
Sex	
Female	10 (53%)
Male	9 (47%)
Race	
Caucasian	15 (79%)
Asian	3 (16%)
African American	1 (5%)
Eastern Cooperative Oncology Group functional status	
0	3 (16%)
1	16 (84%)
Location of pancreatic primary	
Head	11 (58%)
Body or neck	5 (26%)
Tail	3 (16%)
Greatest tumor diameter (cm)	4.0 (2.0–8.9)^a^
Vascular involvement	
Both arterial and venous	10 (53%)
Arterial only	6 (32%)
Venous only	1 (5%)
No vascular involvement	2 (10%)
Lymph node status	
Positive regional nodes on computed tomography (CT) scan	13 (68%)
Negative regional nodes on CT scan	6 (32%)
Serum carbohydrate antigen 19–9 (U/mL)	231 (4–12,414)^a^

^a^ Median (range).

### 3.2 Treatment course

**FIGURE 1:**
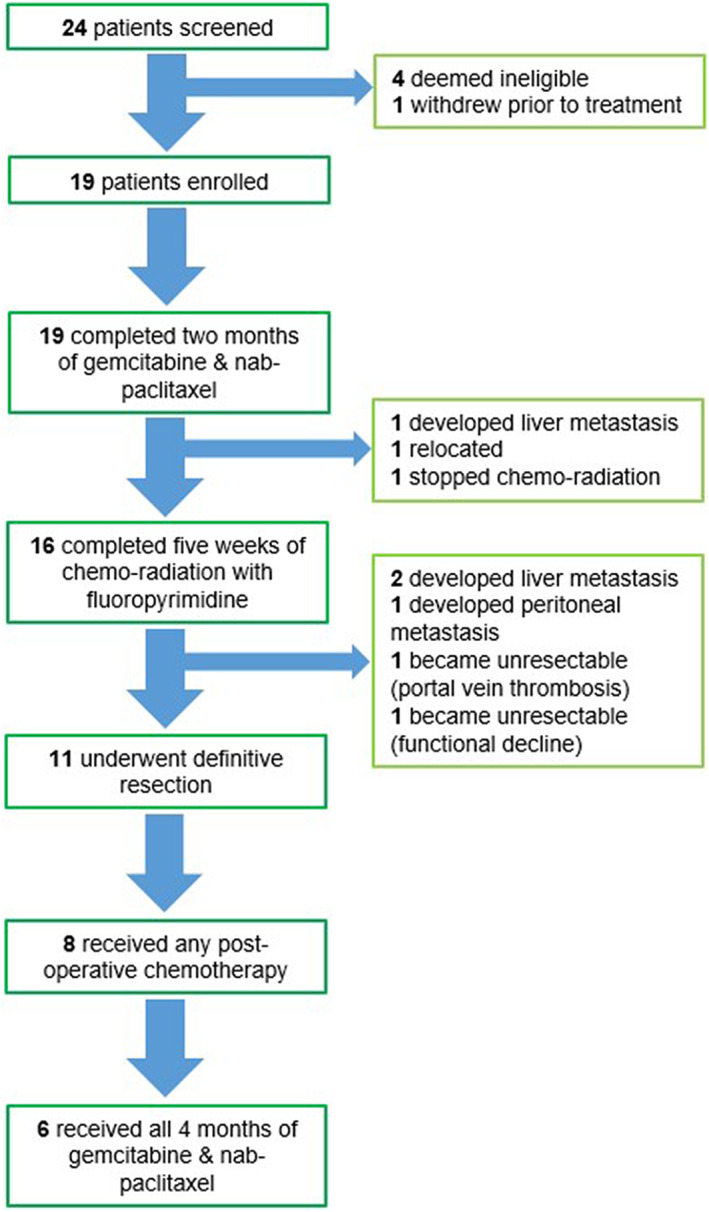
Consort diagram.

All 19 patients received two cycles of gemcitabine and nab‐paclitaxel. One developed rapid CA19‐9 rise and subsequent liver metastasis at first restaging time point after two cycles of chemotherapy. Another one relocated and ended the study prematurely. One proceeded with chemoradiation but was unable to complete it due to functional decline that was retrospectively suspected to be disease progression, but this was unconfirmed. Sixteen patients completed the intended chemoradiation phase of treatment with either 5‐fluorouracil or capecitabine (Figure 1). Following chemoradiation, five patients were unable to proceed further to definitive surgery due to metastatic disease (3), local progression (1), and functional decline (1). Thus, 11 of 19 (58%) underwent definitive resection. Eight patients started postoperative chemotherapy, and only six patients completed all four cycles of postoperative gemcitabine and nab‐paclitaxel, with the majority requiring dose reduction and intermittent dose omissions.

### 3.3 Adverse events related to perioperative therapies

With regard to chemotherapy toxicities, 6 (32%) experienced Grade 3 or 4 neutropenia, two (10%) experienced neutropenic fever, and one developed sepsis during preoperative chemotherapy (Table 2). One patient developed portal vein thrombosis after chemoradiation that may be related to gemcitabine or local progression. Two patients developed functional decline during and after chemoradiation and were unable to proceed to definitive resection. Neutropenia was the most common serious adverse events during postoperative chemotherapy in five of eight participants. One developed duodenal hemorrhage during first cycle of postoperative chemotherapy, but this was attributed as a postoperative complication. Otherwise, all serious adverse events are all detailed in Table 2, and more comprehensive reporting of all adverse events is in Tables S2–S4, which included known side effects of nab‐paclitaxel such as alopecia, nausea, vomiting, fatigue, myalgia, peripheral neuropathy, and rash.

**TABLE 2:** Grade 3 and 4 adverse events during perioperative therapies.

	Counts (%)
Preoperative gemcitabine and nab‐paclitaxel	*N* = 19
Neutropenia	6 (32%)
Febrile neutropenia	2 (11%)
Abdominal pain	1 (5%)
Elevated liver function tests	1 (5%)
Encephalopathy	1 (5%)
Fatigue	1 (5%)
Flank pain	1 (5%)
Hypokalemia	1 (5%)
Portal vein thrombosis	1 (5%)
Sepsis	1 (5%)
Preoperative chemoradiation with fluoropyrimidine	*N* = 17
Lymphopenia and leukopenia	1 (6%)
Postoperative gemcitabine and nab‐paclitaxel	*N* = 8
Neutropenia	5 (62%)
Anemia	1 (13%)
Duodenal hemorrhage	1 (13%)
Elevated liver function tests	1 (13%)
Fatigue	1 (13%)
Hypokalemia	1 (13%)
Lymphopenia and leukopenia	1 (13%)
Nausea	1 (13%)
Sepsis	1 (13%)
Thrombocytopenia	1 (13%)

### 3.4 Surgical events and outcomes

Among the 11 patients who underwent definitive resection, eight (73%) had R0 resection, and three (27%) had R1 resection where the surgical margin was positive microscopically. Six had standard pancreaticoduodenectomy, two had pylorus‐preserving pancreaticoduodenectomy, one had radical antegrade modular pancreaticosplenectomy, one had posterior radical antegrade pancreaticosplenectomy, and one had distal pancreatectomy with splenectomy. Peritoneal deposit was completely resected in one patient, and intraoperative radiation was utilized in another patient. Two patients had a positive margin detected on the superior mesenteric vein margin, and one patient had both positive pancreatic neck and splenic vein margins. Thus, the final R0 resection was eight of 19 (42%, 95% confidence interval [CI] 20%–67%), which met the original criteria for the study to continue beyond Stage 1 to the intended full sample size, before the multidisciplinary team investigators and sponsor terminated the study.

Hospitalization for the 11 resected patients ranged from 6 to 17 days with median of 10 days, with pancreatic leak, ileus, wound infection, and delayed gastric emptying as common reasons for prolonged hospitalizations. Four of 11 patients were readmitted within 30 days (two having failure to thrive, one with delayed gastric emptying, and one with wound infection). One patient was hospitalized six times during the postoperative 90‐day period and ultimately was thought to have radiation enteritis leading to gastrointestinal bleed, pulmonary embolism, failure to thrive, and worsening functional decline, which led to this patient transitioning to hospice. Another patient was rehospitalized within 90 days, but after administration of the first postoperative chemotherapy, for duodenal bleed from gastroduodenal artery stump causing temporary cardiac arrest. This was reversed after resuscitation with massive transfusions and interventional radiology embolization. This patient was able to resume postoperative chemotherapy upon recovery.

### 3.5 Response and survival outcomes

Best radiographic response per RECIST 1.1 following interim contrast CT scans before resection was partial response in 4 of 19 (21%, 95% CI 6%–46%) patients, but stable disease was the more common finding (Table 3). Best pathologic response evaluating resection specimen was near‐complete response in two of 11 (18%, 95% CI 2%–52%) patients, but partial response was the more common finding (Table 3).

**TABLE 3:** Treatment response.

	Counts (%)
Best radiographic response rate (Response Evaluation Criteria in Solid Tumors 1.1)	*N* = 19
Partial response	4 (21%)
Stable disease	14 (74%)
Progressive disease	1 (5%)
Resection outcomes	*N* = 11
R0 resection	8 (73%)
R1 resection	3 (27%)
Pathologic response	*N* = 11
Near‐complete response	2 (18%)
Partial treatment response	9 (82%)

**FIGURE 2:**
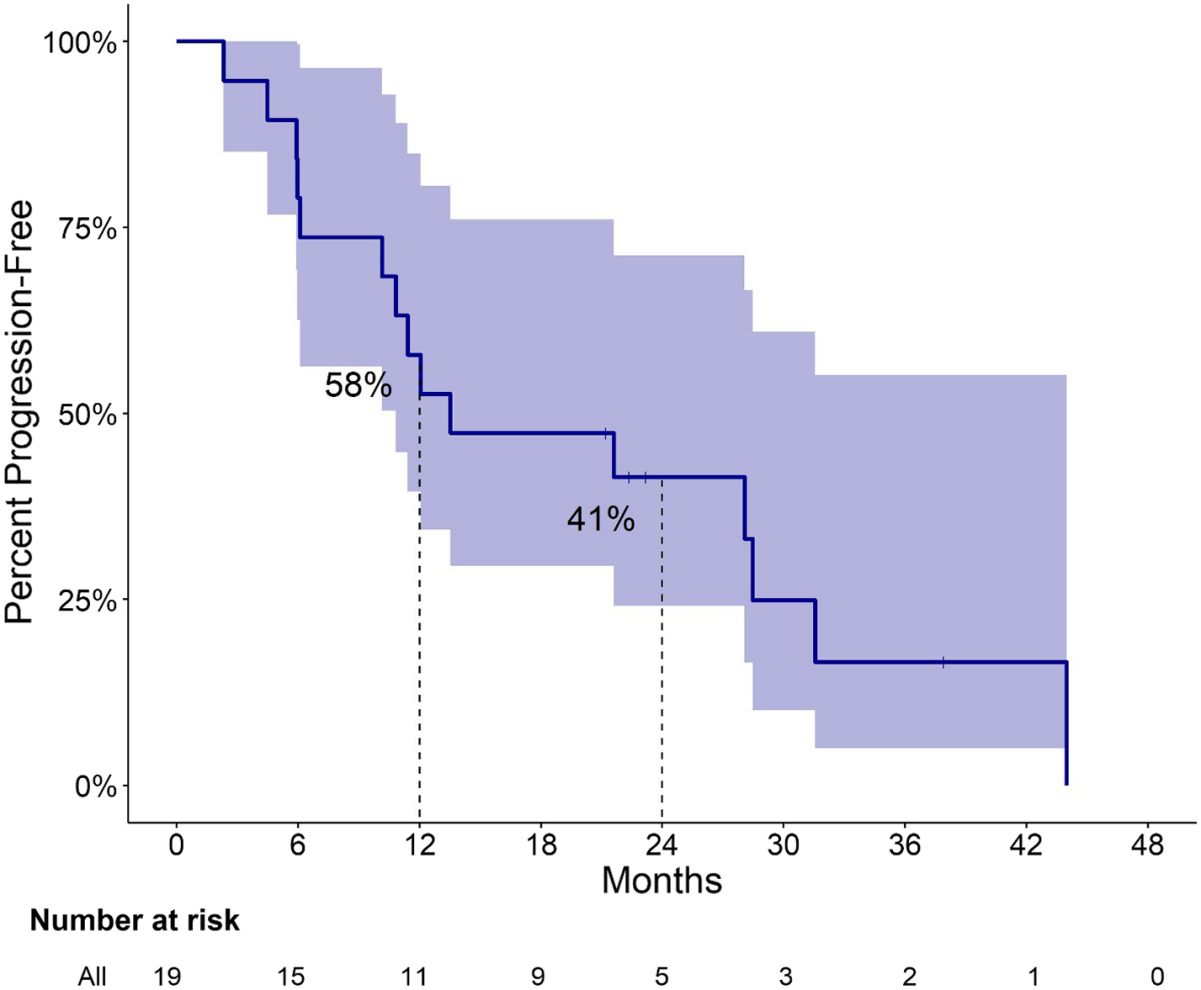
Progression‐free survival analysis from neoadjuvant therapy initiation.

**FIGURE 3:**
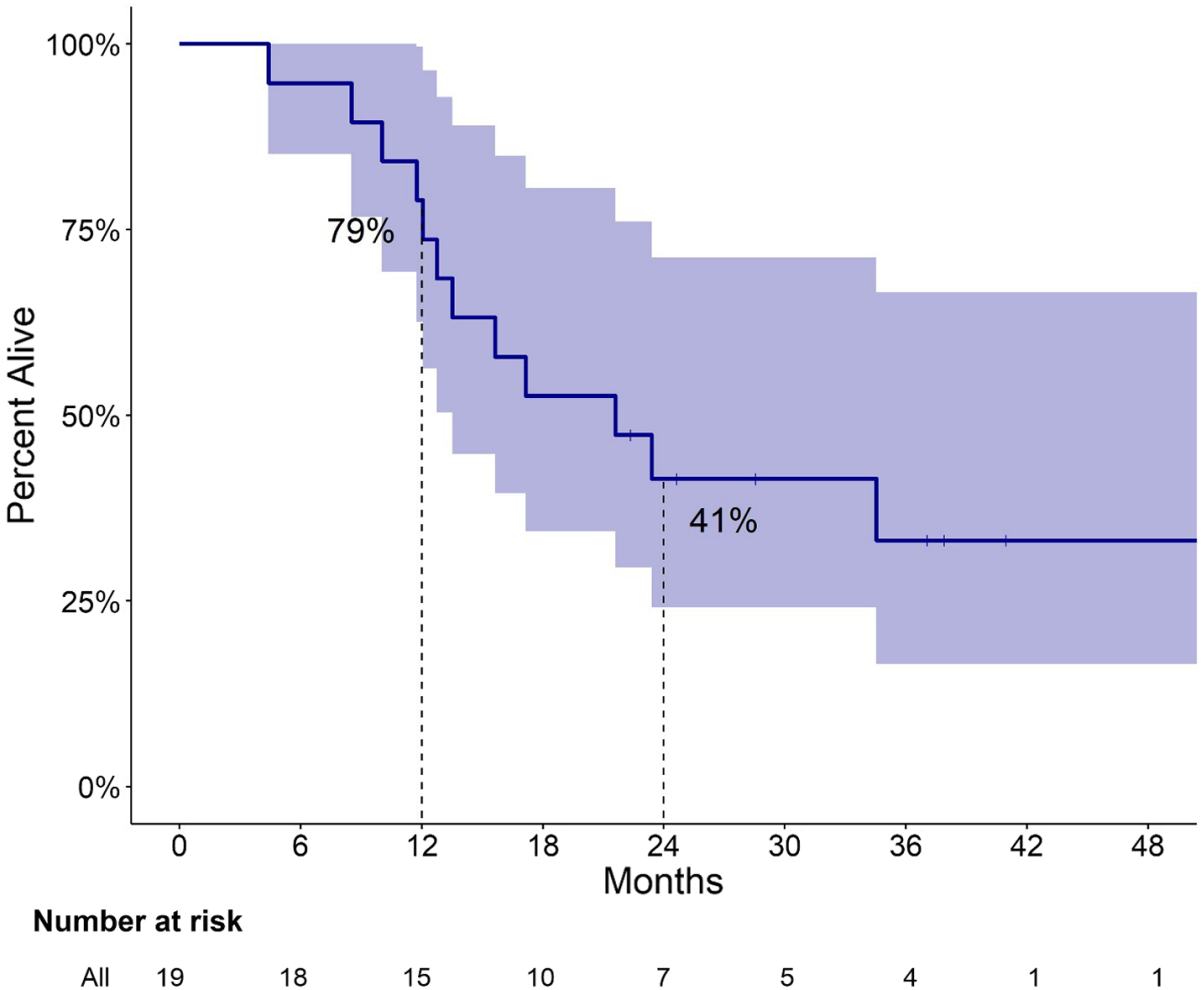
Overall survival analysis from neoadjuvant therapy initiation.

The median duration of follow‐up for all 19 patients was 21.6 (95% CI 13.5–NA) months, with 15 patients having reached an event of interest (e.g., progression and death) at the time of data cutoff. Four remained alive and free of disease at the data cutoff date. The median PFS was 13.5 (95% CI 10.8–NA) months, where the 12‐month PFS was 58% (95% CI 39%–85%; Figure 2). The median overall survival was 21.6 (95% CI 13.5–NA) months, where the 12‐month OS was 79% (95% CI 63%–100%; Figure 3). The 12‐month postsurgery recurrence‐free survival for the 11 patients who underwent definitive resection was 64% (95% CI 41%–99%; Figure S2).

## 4 DISCUSSION

We found that neoadjuvant therapy with nab‐paclitaxel and gemcitabine followed by long‐course chemoradiation with fluoropyrimidine is feasible for patients with borderline resectable and clinically node‐positive pancreatic adenocarcinoma, with a R0 resection of 42% among all 19 participants and a pathologic near‐complete response of 18% among the 11 resection specimens. While both radiographic and pathologic responses were observed from neoadjuvant treatment with chemotherapy and radiation, there is still a high proportion of patients who experienced local and/or metastatic disease progression, some of which resulted in clinical deterioration, prior to or soon after surgical resection. Chemotherapy‐related adverse events such as neutropenia, nausea, fatigue, myalgia, and infections were similar to what is reported in respective package inserts. The population in this study was particularly high risk with 53% of patients having both arterial and venous vascular involvement, thus potentially influencing the number of patients who were ultimately able to undergo resection. Our experience also highlighted the importance of strict evaluation criteria in future studies, such as baseline diagnostic laparoscopy and dedicated CT pancreas protocol to rule out metastatic and unresectable disease that may have been present at baseline.^22^ Even so, our study demonstrated 12‐month PFS of 58% (95% CI 39%–85%) and 12‐month OS of 79% (95% CI 63%–100%) that are similar to survival outcomes from other contemporary neoadjuvant trials, such as PREOPANC, SWOG S1505, and Alliance A021501.^10^, ^14^, ^23^ Ultimately though, patients with vasculature and lymph node involvement have high risk of disease progression and need better therapeutic options to get patients to successful definitive resection.

Several studies have compared neoadjuvant gemcitabine with nab‐paclitaxel to FOLFIRINOX chemotherapy. First, a retrospective cohort of 485 patients at MD Anderson Cancer Center showed that these two neoadjuvant regimens had similar overall survival despite higher resection rates with FOLFIRINOX.^24^ Second, an observational clinical registry from Australia, New Zealand and Singapore, found that those who received FOLFIRINOX had longer overall survival (median 22 vs. 12 months, HR:0.31), but these patients were also younger and had better functional status.^25^ This is in contrast to the recent reporting of the randomized phase 2 trial, SWOG S1505 (NCT02562716), which compared head‐to‐head both regimens given as perioperative strategy in resectable pancreatic cancer, and those treated with gemcitabine and nab‐paclitaxel had 2‐year overall survival of 49% compared to 42% among those treated with FOLFIRINOX.^14^ The overall survival, disease‐free survival, and resection rates were similar between the two treatment arms, but the major pathologic response seemed higher with gemcitabine and nab‐paclitaxel (42% vs. 25%).^14^ All in all, these studies support either neoadjuvant approach for all localized pancreatic cancer without definitively defining a specific treatment regimen as superior over another, which could be further studied.^22^ Neoadjuvant approaches can strategically allow postoperative regimens to adjust based on the pathologic, radiographic, or CA19‐9 biomarker responses to the specific preoperative regimen, as some patients may respond to one regimen and not the other. Notably in Japan, Prep‐02/JSAP05 phase 2/3 trial also demonstrated survival benefit with neoadjuvant gemcitabine and S‐1 chemotherapy over immediate surgery.^26^, ^27^ And finally, the ongoing phase 3 trial in North America, A021806 (NCT04340141), will confirm whether a neoadjuvant approach is truly better than an adjuvant approach for resectable pancreatic cancer, although many clinicians may already be convinced that pancreatic cancer arguably deserves early systemic treatment.^28^

R0 resection is commonly chosen as the primary endpoint among neoadjuvant trials for pancreatic cancer, but such outcomes vary widely among reported trials. A recent analysis from Medical College of Wisconsin showed that when neoadjuvant therapy is specifically given to patients with borderline resectable pancreatic cancer, the expected resection rate is approximately 62% (115 among 185 patients), with final R0 resection rate of 60% (*N* = 111).^29^ As for SWOG S1505 trial, it reported 71% successful resections (73 among 103 eligible patients with resectable pancreatic cancer), with final R0 resection rate of 60% (*N* = 62), though only after excluding 44 ineligible randomized participants.^13^ The surgical outcomes therefore would have been lower in S1505 trial if ineligible patients with metastatic disease and significant vascular invasion were included in the analysis. These studies again echo the importance of detailed evaluation at baseline, as not to create bias and influence the R0 resection endpoint. But other clinical characteristics, such as size of primary tumor, specific vasculature involvement, elevated tumor maker, and positive regional lymph nodes, could also contribute to highly variable R0 resection outcomes from study to study. Furthermore, the duration of neoadjuvant chemotherapy and time from chemoradiation to surgery may influence both the R0 resection and pathologic response findings as well. Interestingly, a recent randomized trial, Alliance A021501 (NCT02839343) suggested that the addition of radiation after neoadjuvant FOLFIRINOX chemotherapy did not always improve R0 resection or survival, and so its clinical application must be considered after multidisciplinary review.^23^ Regardless, a more universally defined patient population and better clinical endpoints need to be further optimized to accurately assess future neoadjuvant therapies.

In the future, pancreatic cancer subtyping by detailed gene expression panels may be able to measure differing responses to chemotherapy and develop clinically relevant blood‐based markers and biopsy‐based markers during a course of neoadjuvant therapy.^30^, ^31^ These biomarker‐based surrogate endpoints may complement survival endpoints and may be used to compare therapeutic agents in neoadjuvant trials. Potential targets within the unique pancreatic tumor microenvironment, from chemokines, to stromal targets, and immune cell interactions, are all urgently needed to improve systemic options and corresponding novel biomarkers in potentially resectable pancreatic cancer.^32^

Several limitations were noted in our study. First, this phase 2 clinical trial which ended prematurely after multidisciplinary review and discussion with the sponsor, and so it did not enroll the intended 44 patients despite meeting interim analysis criteria to continue. Larger trials with randomized design will be needed to confirm efficacy endpoints. Second, diagnostic laparoscopy and dedicated pancreas CT protocol were not required in the original study design, which may have contributed to the lower‐than‐expected resection numbers by possibly including those with occult unresectable and metastatic disease. Third, our neoadjuvant chemotherapy duration of only 2 months of gemcitabine and nab‐paclitaxel is relatively short compared with other contemporary neoadjuvant trials, some of which mandated 4 months of neoadjuvant chemotherapy.^11^ As a result, the surgical outcomes may appear slightly lower than expected, but the overall survival outcomes remained similar to other neoadjuvant trials.

Overall, neoadjuvant therapy with nab‐paclitaxel and gemcitabine along with chemoradiation provides an acceptable R0 resection rate compared with other preoperative treatment regimens. The high risk of distant metastasis in pancreatic cancer highlights the need for detailed baseline workup as well as early, extended multiagent preoperative chemotherapy prior to resection. Several ongoing clinical trials in the Stage 4 setting may identify other neoadjuvant therapy candidates (liposomal irinotecan, NCT04233866; immunotherapy checkpoint inhibitor, NCT03193190). Additionally, an adaptive strategy based on radiographic and blood‐based assessments, recently activated at our institution, could yield optimal clinical outcomes in potentially resectable patients receiving neoadjuvant therapies (NeoOPTIMIZE, NCT04539808). Therefore, preoperative gemcitabine and nab‐paclitaxel‐based chemotherapy, perhaps of longer duration than two cycles, followed by chemoradiation represents a feasible, alternate strategy to FOLFIRINOX‐based therapy for borderline resectable pancreatic cancer, especially for those patients not felt to be candidates for more intensive regimens. Ultimately, both regimens could be personalized for future patients with potentially resectable pancreatic cancer.

## AUTHOR CONTRIBUTIONS

**Emerson Chen:** Data curation (equal); formal analysis (equal); investigation (equal); visualization (equal); writing – original draft (lead); writing – review and editing (equal). **Adel Kardosh:** Data curation (equal); formal analysis (supporting); investigation (equal); writing – review and editing (supporting). **Nima Nabavizadeh:** Formal analysis (supporting); investigation (equal); writing – review and editing (supporting). **Bryan Foster:** Conceptualization (supporting); data curation (supporting); investigation (supporting); methodology (supporting); writing – review and editing (supporting). **Skye Mayo:** Conceptualization (supporting); formal analysis (supporting); methodology (supporting); writing – review and editing (supporting). **Kevin G. Billingsley:** Conceptualization (equal); methodology (equal); supervision (equal); writing – review and editing (equal). **Erin W. Gilbert:** Conceptualization (supporting); writing – review and editing (supporting). **Christian Lanciault:** Conceptualization (supporting); data curation (supporting); investigation (supporting); methodology (supporting); writing – review and editing (supporting). **Aaron Grossberg:** Investigation (supporting); writing – review and editing (supporting). **Kenneth G. Bensch:** Data curation (supporting); writing – review and editing (supporting). **Erin Maynard:** Data curation (supporting); writing – review and editing (supporting). **Eric C. Anderson:** Conceptualization (equal); data curation (equal); investigation (equal); methodology (equal); writing – review and editing (supporting). **Brett Sheppard:** Conceptualization (equal); methodology (equal); resources (equal); supervision (equal); writing – review and editing (equal). **Charles R. Thomas:** Conceptualization (equal); formal analysis (equal); methodology (equal); project administration (equal); resources (equal); supervision (equal); writing – review and editing (equal). **Charles D Lopez:** Conceptualization (equal); formal analysis (equal); funding acquisition (equal); investigation (equal); methodology (equal); project administration (equal); resources (equal); supervision (equal); writing – review and editing (equal). **Gina M. Vaccaro:** Conceptualization (equal); formal analysis (supporting); funding acquisition (lead); investigation (equal); methodology (equal); project administration (lead); resources (equal); supervision (lead); writing – original draft (supporting); writing – review and editing (lead).

## FUNDING INFORMATION

OHSU Knight Cancer Institute, Celgene.

## CONFLICT OF INTEREST STATEMENT

Emerson Y. Chen has received honorarium from Horizon CME and has provided research support for Taiho Oncology. Adel Kardosh has provided consulting support for Exelixis, Astellas Pharma, Astex Pharmaceuticals, and Genetech, and research support for Natera. Bryan Foster, Skye C. Mayo, Kevin G. Billingsley, Erin W. Gilbert, Christian Lanciault, Aaron Grossberg, Kenneth G. Bensch, Erin Maynard, and Brett C. Sheppard have no conflicts of interest. Eric C. Anderson has provided consulting support for Roche/Genetech and Aileron Therapeutics. Charles R. Thomas Jr. has provided consulting support for AMA and F3 Platform Biologics. Charles D. Lopez has obtained research funding from Roche/Genentech, and has provided research support for Taiho Oncology. Gina M. Vaccaro has obtained research funding from Celgene.

## ETHICS STATEMENT

All research activities were approved by OHSU institutional review board (IRB).

## PATIENT CONSENT

Written consented was obtained from all participants of clinical trial.

## Supporting information


Figure S1.
Figure S2.
Table S1.
Table S2.
Table S3.
Table S4.

## Data Availability

Data Availability: upon request and review of OHSU investigators.
