# Supplementary material for: Phase 2 study of preoperative chemotherapy with nab‐paclitaxel and gemcitabine followed by chemoradiation for borderline resectable or node‐positive pancreatic ductal adenocarcinoma
Source: Cancer Med. 2023 May 3;12(12):12986–95. doi: 10.1002/cam4.5971 (PMC10315770; doi:10.1002/cam4.5971)

**Supplement**

**Figure S1** Study Schema ……………………………………………………………………….1

**Table S1** Dose Modification Scheme …………………………………………………………..2

**Table S2** Selected adverse reactions during pre-operative chemotherapy ….………………….3

**Table S3** Selected adverse reactions during pre-operative chemo-radiation ……………….…..5

**Table S4** Selected adverse reactions during post-operative chemotherapy ...…………….….…6

**Figure S2** Post-surgery recurrence-free survival ……………………………………………….7

**Figure S1 Study Schema**

**
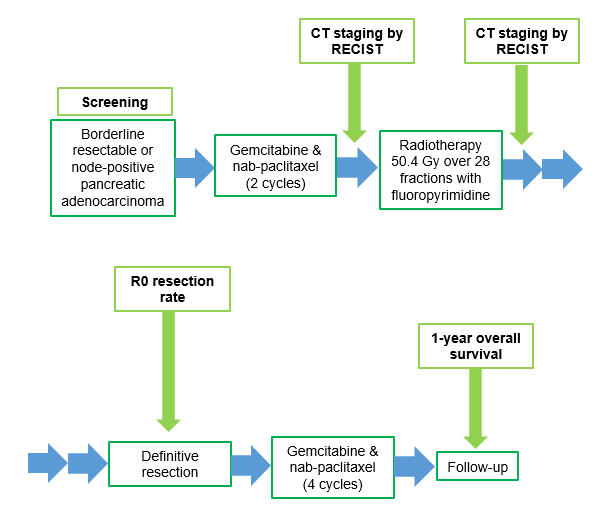
**

**Table S1 Dose Modification Scheme**

Day 1 of each cycle ANC must be ≥ 1500 and Platelets ≥ 100,000 to proceed with chemotherapy

| **Dose Level** | **Nab-paclitaxel (mg/m^2^)** | **Gemcitabine (mg/m^2^)** |
| --- | --- | --- |
| Full dose | 125 | 1000 |
| 1^st^ dose reduction | 100 | 800 |
| 2^nd^ dose reduction | 75 | 600 |
| If additional dose reduction required | Discontinue | Discontinue |

Dose modifications for Neutropenia and/or Thrombocytopenia at the start or within a cycle:

| **Cycle Day** | **ANC (cells/mm^3^)** |  | **Platelet count (cells/mm^3^)** | **Nab-paclitaxel/ Gemcitabine** |
| --- | --- | --- | --- | --- |
| Day 1 | < 1500 | OR | < 100,000 | Delay doses until recovery |
| Day 8 | 500 to < 1000 | OR | 50,000 to < 75,000 | Reduce 1 dose level |
|  | < 500 | OR | < 50,000 | Withhold doses |
| Day 15: If Day 8 doses were reduced or given without modification: | | | | |
|  | 500 to < 1000 | OR | 50,000 to < 75,000 | Reduce 1 dose level from Day 8 |
|  | < 500 | OR | < 50,000 | Withhold doses |
| Day 15: If Day 8 doses were withheld: | | | | |
|  | ≥ 1000 | OR | ≥ 75,000 | Reduce 1 dose level from Day 1 |
|  | 500 to < 1000 | OR | 50,000 to < 75,000 | Reduce 2 dose levels from Day 1 |
|  | < 500 | OR | < 50,000 | Withhold doses |

Dose Modifications for Non-Hematologic Toxicity:

| **Toxicity** | **ABRAXANE** | **Gemcitabine** |
| --- | --- | --- |
| Febrile Neutropenia  (Grade 3 or 4) | Withhold until fever resolves and ANC ≥ 1500: resume at next lower dose level | |
| Peripheral Neuropathy  (Grade 3 or 4) | Withhold until improves to ≤ Grade 1; resume at next lower dose level | No dose reduction |
| Cutaneous Toxicity  (Grade 2 or 3) | Reduce to next lower dose level; discontinue treatment if toxicity persists | |
| Gastrointestinal Toxicity  (Grade 3 mucositis or diarrhea) | Withhold until improves to ≤ Grade 1; resume at next lower dose level | |

Dosing re-escalation is allowed at the provider’s discretion

**Table S2.** Selected adverse reactions with higher incidence (>10%) or notable from package insert (nab-paclitaxel) during pre-operative gemcitabine & nab-paclitaxel therapy per CTCAE terminology version 4.0. **N=19**

| **System Organ Class** | **Adverse Reaction** | **Grade 1 or 2** | **Grade 3 or 4** |
| --- | --- | --- | --- |
| Blood and lymphatic system disorders | Anemia | 2 (11%) | - |
|  | Neutropenic fever | - | 2 (11%) |
| Gastrointestinal disorders | Abdominal/flank pain | 1 (5%) | 1 (5%) |
|  | Constipation | 4 (21%) | - |
|  | Diarrhea | 3 (16%) | - |
|  | Oral mucositis | 1 (5%) | - |
|  | Nausea | 8 (42%) | - |
|  | Vomiting | 6 (32%) | - |
| General disorders and administration site conditions | Chills | 3 (16%) | - |
|  | Edema limbs | 4 (21%) | - |
|  | Fatigue | 7 (37%) | 1 (5%) |
|  | Fever | 4 (21%) | - |
|  | Flu-like symptoms | 4 (21%) | - |
|  | Non-cardiac chest pain | 2 (11%) | - |
| Hepatobiliary disorders | Portal vein thrombosis | - | 1 (5%) |
| Infections and infestations | Sepsis | - | 1 (5%) |
|  | Skin infection | 1 (5%) | - |
|  | Lung infection | 1 (5%) | - |
| Investigations | Elevated liver function tests | 1 (5%) | 1 (5%) |
|  | Neutropenia | 1 (5%) | 6 (32%) |
|  | Thrombocytopenia | 4 (21%) | - |
|  | Leukopenia | 1 (5%) | 1 (5%) |
| Metabolism and nutrition disorders | Anorexia | 2 (11%) | - |
|  | Dehydration | 1 (5%) | - |
|  | Hypokalemia | 2 (11%) | 1 (5%) |
| Musculoskeletal and connective tissue disorders | Arthralgia | 2 (11%) | - |
|  | Asthenia | 1 (5%) | - |
|  | Back pain | 3 (16%) | - |
|  | Bone pain | 2 (11%) | - |
|  | Flank pain | - | 1 (5%) |
|  | Myalgia | 5 (26%) | - |
| Nervous system disorders | Dizziness | 1 (5%) | - |
|  | Dysgeusia | 3 (16%) | - |
|  | Encephalopathy | - | 1 (5%) |
|  | Headache | 1 (5%) | - |
|  | Peripheral sensory neuropathy | 8 (42%) | - |
|  | Transient ischemic attack | 1 (5%) | - |
| Psychiatric disorders | Confusion | 1 (5%) | - |
|  | Depression | 1 (5%) | - |
| Respiratory, thoracic and mediastinal disorders | Dyspnea | 3 (16%) | - |
|  | Epistaxis | 4 (21%) | - |
| Skin and subcutaneous tissue disorders | Acneiform rash | 2 (11%) | - |
|  | Alopecia | 9 (47%) | - |
|  | Maculopapular rash | 7 (37%) | - |
|  | Palmar-plantar erythrodyesthesia | 1 (5%) | - |
| Vascular disorders | Hypertension | 2 (11%) | - |
|  | Superficial thrombophlebitis | 1 (5%) | - |

*The following adverse reactions were grade 1 or 2, and were only recorded in 1 participant: dry mouth, dry skin, dyspepsia, hot flashes, hypoalbuminemia, insomnia, lethargy, localized edema, generalized muscle weakness, sinus tachycardia, sore throat, thrush, urticaria, weight loss, vascular access complication, elevated pancreatic enzyme, and gastroesophageal reflux.

**Table S3.** Selected adverse reactions with higher incidence (>10%) or notable during pre-operative chemo-radiation with fluoropyrimidine per CTCAE terminology version 4.0. **N=17**

| **System Organ Class** | **Adverse Reaction** | **Grade 1 or 2** | **Grade 3 or 4** |
| --- | --- | --- | --- |
| Blood and lymphatic system disorders | Anemia | 1 (6%) | - |
| Ear and labyrinth disorders | Vertigo | 2 (12%) | - |
| Gastrointestinal disorders | Abdominal pain | 1 (6%) | - |
|  | Constipation | 2 (12%) | - |
|  | Diarrhea | 4 (24%) | - |
|  | Gastritis | 1 (6%) | - |
|  | Oral mucositis | 3 (18%) | - |
|  | Nausea | 4 (24%) | - |
|  | Vomiting | 5 (29%) | - |
| General disorders and administration site conditions | Fatigue | 5 (29%) | - |
| Infections and infestations | Skin infection | 1 (6%) | - |
|  | Upper respiratory infection | 1 (6%) | - |
| Investigations | Leukopenia | - | 1 (6%) |
|  | Lymphopenia | - | 1 (6%) |
|  | Neutropenia | 1 (6%) | - |
|  | Thrombocytopenia | 1 (6%) | - |
| Metabolism and nutrition disorders | Anorexia | 4 (24%) | - |
|  | Dehydration | 3 (18%) | - |
| Nervous system disorders | Dysgeusia | 1 (6%) | - |
|  | Dizziness | 1 (6%) | - |
|  | Peripheral sensory neuropathy | 1 (6%) | - |
| Respiratory, thoracic and mediastinal disorders | Cough | 2 (12%) | - |
| Skin and subcutaneous tissue disorders | Alopecia | 1 (6%) | - |
|  | Palmar-plantar erythrodyesthesia | 1 (6%) | - |
|  | Maculopapular rash | 1 (6%) | - |

*The following adverse reactions were grade 1 or 2, and were only recorded in 1 participant: cognitive disturbance, conjunctivitis, dyspepsia, epistaxis, gastritis, gout, lethargy, pain in extremity, pain of skin, productive cough, urinary intention, erectile dysfunction, generalized muscle weakness, and vascular access complication.

**Table S4.** Selected adverse reactions notable from package insert (nab-paclitaxel) during post-operative gemcitabine & nab-paclitaxel therapy per CTCAE terminology version 4.0. **N=8**

| **System Organ Class** | **Adverse Reaction** | **Grade 1 or 2** | **Grade 3 or 4** |
| --- | --- | --- | --- |
| Blood and lymphatic system disorders | Anemia | 1 (13%) | 1 (13%) |
| Gastrointestinal disorders | Constipation | 2 (25%) | - |
|  | Duodenal hemorrhage | - | 1 (13%) |
|  | Diarrhea | 3 (38%) | - |
|  | Oral mucositis | 1 (13%) | - |
|  | Nausea | 1 (13%) | 1 (13%) |
|  | Vomiting | 2 (25%) | - |
| General disorders and administration site conditions | Edema limbs | 2 (25%) | - |
|  | Fatigue | 2 (25%) | 1 (13%) |
| Infections and infestations | Anemia | 1 (13%) |  |
|  | Sepsis | - | 1 (13%) |
| Investigations | Elevated liver function tests | 1 (13%) | 1 (13%) |
|  | Neutropenia | 1 (13%) | 5 (62%) |
|  | Thrombocytopenia | 1 (13%) | 1 (13%) |
|  | Leukopenia | - | 1 (13%) |
|  | Lymphopenia | 1 (13%) | 1 (13%) |
| Metabolism and nutrition disorders | Anorexia | 2 (25%) | - |
| Metabolism and nutrition disorders | Hyperglycemia | 1 (13%) | - |
|  | Hypoalbuminemia | 1 (13%) | - |
|  | Hypokalemia | - | 1 (13%) |
| Musculoskeletal and connective tissue disorders | Asthenia | 2 (25%) | - |
| Nervous system disorders | Dysgeusia | 1 (13%) | - |
|  | Headache | 1 (13%) | - |
|  | Peripheral sensory neuropathy | 2 (25%) | - |
| Respiratory, thoracic and mediastinal disorders | Dyspnea | 2 (25%) | - |
| Skin and subcutaneous tissue disorders | Alopecia | 2 (25%) | - |
|  | Acneiform rash | 1 (13%) | - |
|  | Maculopapular rash | 2 (25%) | - |

*The following adverse reactions were grade 1 or 2, and were only recorded in 1 participant: bone pain, epistaxis, flank pain, joint effusion, nasal congestion, skin hyperpigmentation, mitral regurgitation, generalized muscle weakness, and urinary tract pain.

**Figure S2 Post-surgery recurrence-free survival**


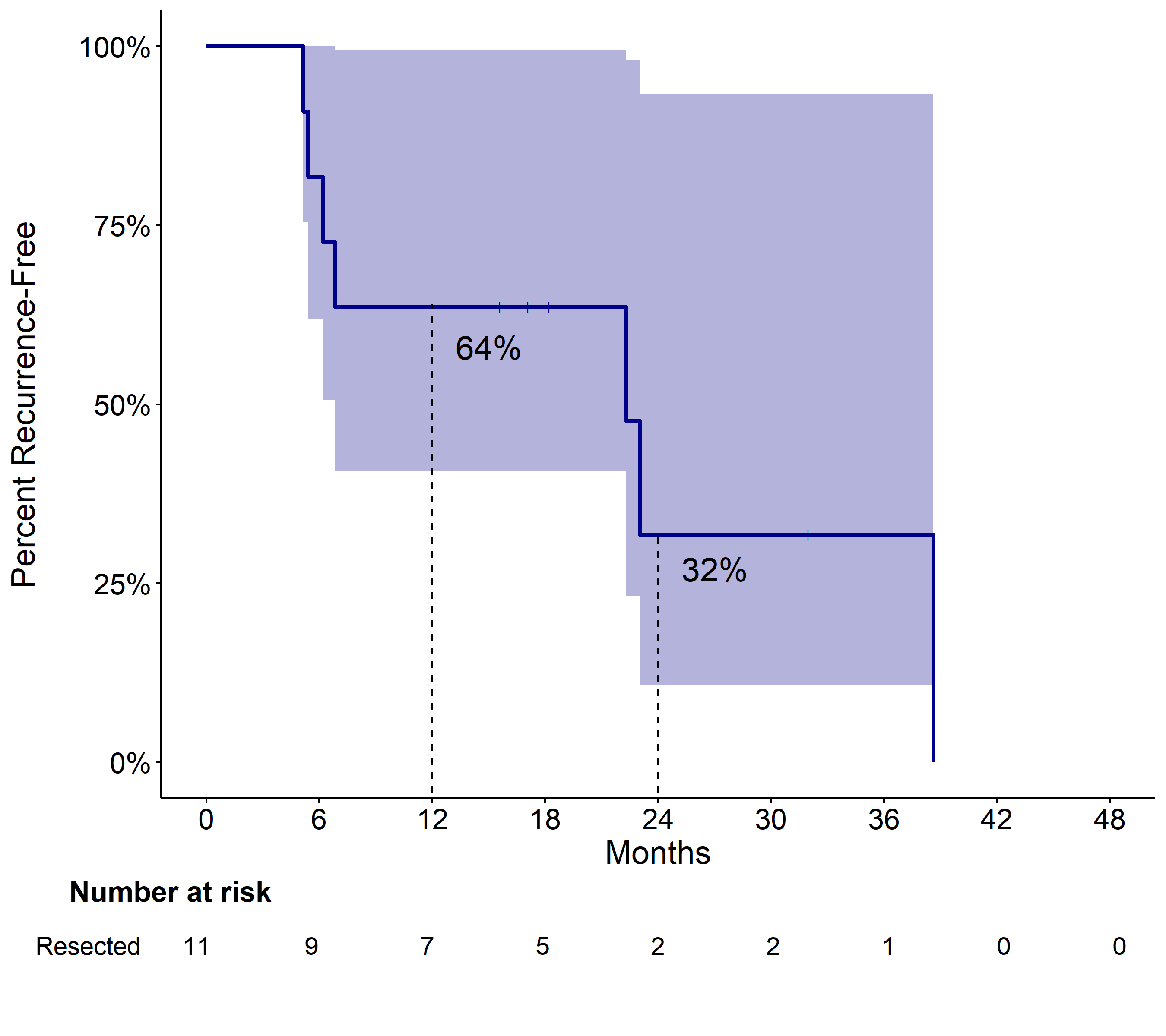

Supplement: Supplementary file 1 — Figure S1. Figure S2. Table S1. Table S2. Table S3. Table S4. [file CAM4-12-12986-s001.docx]
